# Structural basis for broad-spectrum binding of AT-9010 to flaviviral methyltransferases

**DOI:** 10.1007/s00705-025-06227-3

**Published:** 2025-02-20

**Authors:** Katerina Krejcova, Evzen Boura

**Affiliations:** Institute of Organic Chemistry and Biochemistry, Academy of Sciences of the Czech Republic, v.v.i, Flemingovo nám. 2, Prague, 16610 Czech Republic

## Abstract

AT-9010 (2'-methyl-2'-fluoro guanosine triphosphate) is a GTP analog whose prodrug, AT-752 is under consideration in human medicine as a potential antiviral drug against certain flaviviruses. It was previously believed to inhibit viral replication by acting primarily as a chain terminator. However, it was discovered recently that it also binds the GTP binding site of the methyltransferase (MTase) domain of the orthoflavivirus polymerase, thus interfering with RNA capping. Here, we investigated the binding of AT-9010 to Ntaya and Zika virus MTases. Structural analysis using X-ray crystallography revealed similar interactions between the base and sugar moieties of AT-9010 and key residues in both MTases, although differences in hydrogen bonding were observed. Our analysis also suggested that the triphosphate part of AT-9010 is flexible. Despite minor variations, the overall binding mode of AT-9010 was found to be the same for all of the flaviviral MTases examined, suggesting a structural basis for the efficacy of AT-9010 against multiple orthoflavivirus MTases.

**Supplementary Information:**

The online version contains supplementary material available at 10.1007/s00705-025-06227-3.

## Introduction

The family *Flaviviridae* includes four genera: *Orthoflavivirus*, *Hepacivirus*, *Pegivirus*, and *Pestivirus* [1]. The NS5 protein of members of the genus *Orthoflavivirus* is composed of two domains: an N-terminal methyltransferase (MTase) domain and a C-terminal RNA-dependent RNA polymerase (RdRp) domain. The RdRp is a well-described and established antiviral target [2–5], and several compounds targeting orthoflaviviral RdRps have been described [6–8], including many nucleotide triphosphate analogs such as remdesivir triphosphate and AT-9010 [9–11].

The orthoflaviviral MTase domain binds to GTP and acts as a guanylyltransferase, and it also binds to S-adenosyl methionine (SAM) and catalyzes the transfer of a methyl group to the N7 position of the pre-cap structure and to the 2′-O position of the penultimate nucleotide of the capped 5′ end [12]. These catalytic activities are important for viral replication, and the MTase domain is therefore a potential target for antiviral drugs [13, 14].

The development of inhibitors against viral MTases started more than a decade ago [15, 16], and the MTases of the most medically important flaviviruses were characterized before the COVID-19 pandemic [3, 4, 17]. However, during the COVID-19 pandemic, MTases became the focus of intense scientific scrutiny when the MTases of SARS-CoV-2 were characterized and specific inhibitors of these enzymes were developed [18–23]. During the same period, the MTases of other unrelated viruses, including Mpox virus, were also characterized, and inhibitors were developed against these enzymes [24–26]. Later, during and after the pandemic, the crystal structures of flaviviral MTases from lesser-known flaviviruses such as Usutu, Langat, and Ntaya viruses also became available [27–29]. Interestingly, the catalytic tetrad responsible for the catalysis of 2′-O methylation is absolutely conserved among otherwise unrelated viruses such as coronaviruses, flaviviruses, and poxviruses [30, 31]. In contrast, the GTP binding sites of their capping enzymes show no resemblance (Supplementary Fig. S1).

Most of the MTase inhibitors target the SAM or RNA binding site, with the exception of AT-9010, which is a fluorinated analog of GTP (2'-methyl-2'-fluoro guanosine triphosphate). The presence of the fluorine atom, instead of a 2′ hydroxyl group, in the ribose ring inevitably leads to the termination of RNA replication when a non-proofreading RNA-dependent RNA polymerase (RdRp) uses it instead of a GTP molecule. Notably, while most viral RdRps lack proofreading activity, in members of the order *Nidovirales*, the replication complex includes an exonuclease that enables proofreading [32].

As a triphosphate, AT-9010 cannot cross the plasma membrane. To overcome this limitation, the prodrugs AT-752 and AT-281 have been designed. Both of these prodrugs have low cytotoxicity and are converted to the active triphosphate form within cells through a series of enzymatic reactions [33]. AT-281 and AT-752 have been shown to be effective against flaviviruses in cell culture [34]. AT-752 also gave promising results when tested in a hamster model of yellow fever [35] and was shown to be non-toxic in humans [36]. Due to its chemical structure, it was believed that the sole target of AT-9010 is the RdRp. However, it was recently shown that it also binds to the GTP binding site of the methyltransferase (MTase) domain of the flaviviral RdRp polymerase NS5 [34]. In this study, we investigated the binding of AT-9010 to other flaviviral MTases to assess whether the binding mode of AT-9010 is conserved among flaviviruses. We selected the MTase domains from Zika and Ntaya viruses. Zika virus, which caused an epidemic during the 2016 Summer Olympic Games in Brazil [37], was first identified in the 1950s and was not initially considered highly dangerous to humans [38]. Ntaya virus was also discovered in the 1950s [39] and is also a mosquito-borne virus. While Ntaya virus is known to be transmissible to humans and capable of causing febrile illness, it is not considered particularly dangerous [40]. In this study, we determined the X-ray crystal structures of the MTase domains of both of these viruses in complex with AT-9010 and compared the binding modes with that reported previously for AT-9010 in complex with the dengue virus MTase.

## Materials and methods

### Protein expression and purification

Genes encoding the Zika and Ntaya MTase domains were artificially synthesized (Thermo Fisher Scientific) and cloned into the vector pSUMO, which we described previously [41], so that the recombinant protein contained an N-terminal 8x-His-SUMO solubilisation/purification tag. Proteins were expressed and purified as described previously [29, 42]. Briefly, the genes for Ntaya and Zika virus MTase were expressed in *E. coli* BL21-CodonPlus (DE3) RIL in LB medium. The bacteria were harvested and resuspended in lysis buffer containing 50 mM Tris, pH 8.0, 500 mM NaCl, 20 mM imidazole, 10% (v/v) glycerol, and 3 mM β-mercaptoethanol. The supernatant was then immobilized on Ni-NTA agarose beads (Machery-Nagel) and eluted using lysis buffer supplemented with 300 mM imidazole. Subsequently, the 8x-His-SUMO tag was cleaved by the Ulp1 protease at 4°C overnight while dialyzing against the lysis buffer. The 8x-His-SUMO tag and any traces of uncleaved protein were separated using Ni-NTA agarose beads. Subsequently, the proteins were purified by size exclusion chromatography (SEC) using Superdex 75 16/600 (GE Life Sciences) running in SEC buffer (25 mM HEPES, pH 7.5, 500 mM NaCl, 5% (v/v) glycerol, and 1 mM Tris(2-carboxyethyl)phosphine [TCEP]). Finally, the proteins were concentrated to 10 mg/ml and used for crystallization trials or stored at -80°C until needed.

### Crystallization and crystallographic analysis

Crystals of Ntaya and Zika virus MTase domains grew for 4 days at 18°C in sitting drops. The proteins were mixed 1:1 with the well solution. The well solution for the Ntaya virus protein consisted of 0.2 M sodium acetate trihydrate, 0.1 M sodium HEPES, pH 7.5, 25% (w/v) polyethylene glycol (PEG) 3350, and for the Zika virus protein, it consisted of 0.2 M MgCl_2_, 0.1 M HEPES, pH 7.5, 25% (w/v) PEG 3350.

Crystals of MTases were then soaked overnight with 10 mM AT-9010 in the presence of 1 mM Mg^2+^. The soaked crystals were then cryoprotected in well solution supplemented with 20% (v/v) glycerol and flash frozen in liquid nitrogen. The crystals of both the Zika and Ntaya virus MTase domains belonged to the P2_1_ spacegroup and diffracted to 2 Å and 1.8 Å, respectively.

The datasets were collected using our in-house X-ray source (rotating anode, Rigaku micromax-007 HF). The data were integrated and scaled using XDS [43]. The structures were solved by molecular replacement, using the structures of Ntaya virus MTase (pdb entry: 8QDJ) [29] and Zika virus MTase (PDB entry 5MRK) [42]. The initial models were obtained using Phaser in the Phenix package [44]. The models were further improved using automatic model refinement with Phenix.refine, followed by manual model building with Coot [45]. Statistics for data collection and processing and structure determination and refinement are summarized in Table 1. Structural images were generated using PyMOL Molecular Graphics System v2.0 (Schrödinger, LLC). The atomic coordinates and structural factors were deposited in the Protein Data Bank (https://www.rcsb.org).

**Table 1:** Data-collection and processing statistics. Values in parentheses are for the highest-resolution shell

Crystal	Zika MTase + AT-9010	Ntaya MTase + AT-9010
PDB accession code	8PEM	9GJZ
**Data collection and processing**		
Space group	P2_1_	P2_1_
Cell dimensions - a, b, c (Å)	39.56, 41.02, 69.03	38.33, 71.58, 50.33
Cell dimensions - α, β, γ (°)	90.00, 96.25, 90.00	90, 92.64, 90
Resolution range (Å)	28.38–2 (2.074– 2.002)	35.79 − 1.74 (1.81 − 1.74)
No. of unique reflections	14679 (1430)	49191 (5018)
Completeness (%)	97.20(94.75)	95.24 (87.59)
Multiplicity	3.2 (3.2)	1.8 (1.8)
Mean I/σ(I)	4.90 (1.24)	6.87 (1.32)
CC_1/2_	0.975 (0.385)	0.991 (0.443)
CC*	0.994 (0.746)	0.998 (0.784)
**Structure solution and refinement**		
R-work (%)	22.82 (30.60)	17.28 (26.16)
R-free (%)	26.21 (32.47)	20.03 (27.35)
R.m.s.d. - bonds (Å) / angles (°)	0.003 / 0.69	0.006 / 0.86
Average B factors (Å^2^)	21.65	18.64
Protein	21.39	17.02
Ligand	36.96	24.79
Solvent	21.58	27.44
Clash score	2.5	3.98
Ramachandran favored/outliers (%)	98.05 / 0	98.85 / 0

## Results

**Fig. 1:**
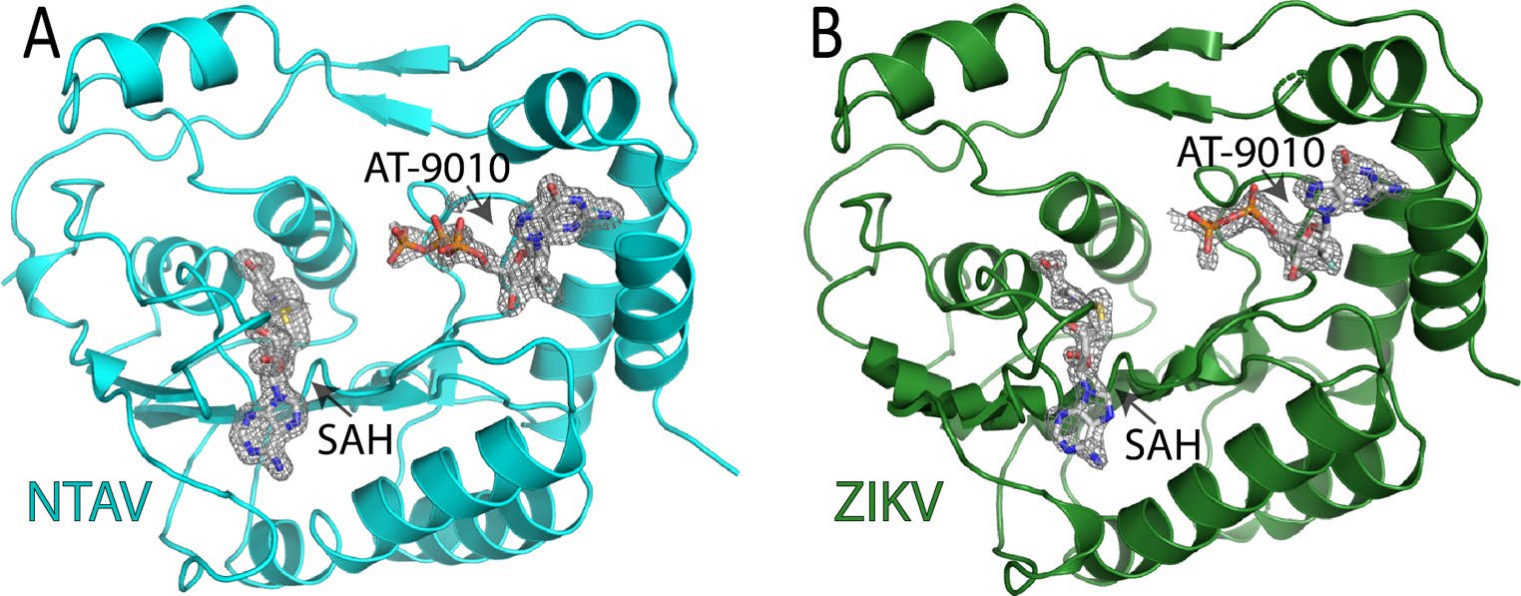
Crystal structure of Ntaya and Zika virus MTase domains in complex with AT-9010 and SAH. (**A**) Overall structure of the Ntaya virus MTase domain with bound AT-9010 and SAH. The Fo-Fc omit electron density maps contoured at 3σ are displayed for the AT-9010 and SAH molecules. (**B**) Overall structure of the Zika virus MTase domain with bound AT-9010 and SAH. The Fo-Fc omit electron density map is contoured at 3σ for the SAH molecule and at 2σ for AT-9010

We prepared crystals of Ntaya and Zika virus MTases as described previously [29, 42] and soaked them overnight with 10 mM AT-9010. The crystals were expected to contain S-adenosyl homocysteine (SAH) in addition to AT-9010, as recombinant flaviviral MTases co-purify with SAH from bacteria [46]. The structures were determined by molecular replacement, and the electron density for both expected ligands, AT-9010 and SAH, was clearly visible. The AT-9010 molecule was in both cases located in the GTP binding site, as expected. However, the electron density was better defined for the AT-9010 molecule that was bound to the Ntaya virus MTase (Fig. 1). In fact, in the case of the Zika virus MTase, we did not observe any density corresponding to the γ-phosphate of AT-9010, and we therefore did not model it.

**Fig. 2:**
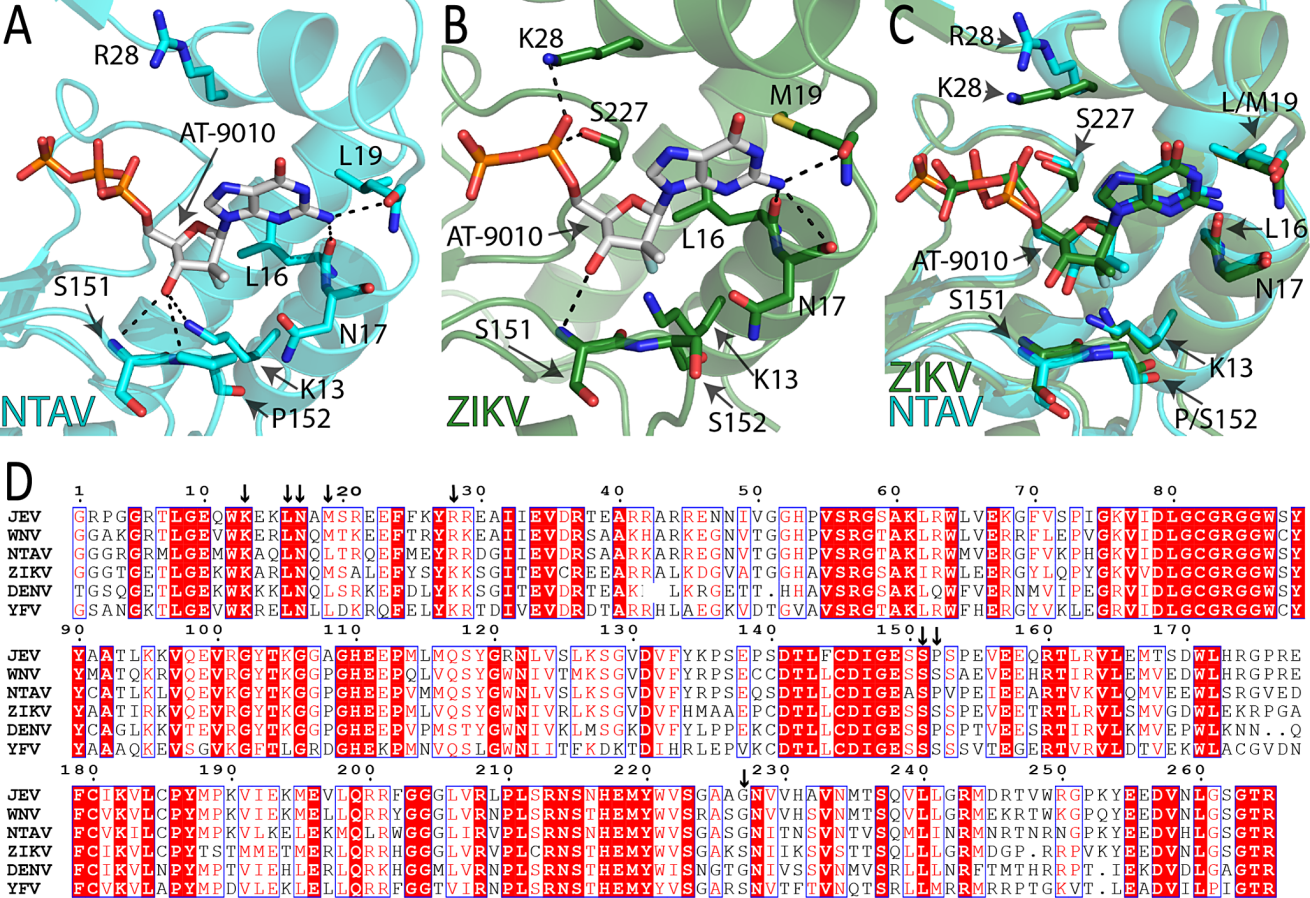
Binding of AT-9010 to the Ntaya and Zika virus MTase domains. (**A**) A detailed view of residues of the GTP binding site of the Ntaya virus MTase domain that interact with AT-9010. Hydrogen bonds are depicted, and key residues are labeled. (**B**) A detailed view of AT-9010 bound to residues of the GTP binding site of the Zika virus MTase domain that interact with AT-9010. Hydrogen bonds are depicted, and key residues are labeled. (**C**) Structural alignment of the AT-9010 binding sites of the Ntaya virus (cyan) and Zika virus (green) MTase domains. The phosphorus atoms of AT-9010 in the Ntaya virus MTase structure are colored orange, and those in the Zika virus MTase structure are colored green. Key residues are labeled. (**D**) Primary sequence alignment of selected flaviviral MTase domains. Conserved residues are highlighted in red. The alignment was generated using the ESPript 3.0 online program (https://espript.ibcp.fr/ESPript/ESPript/)

**Fig. 3:**
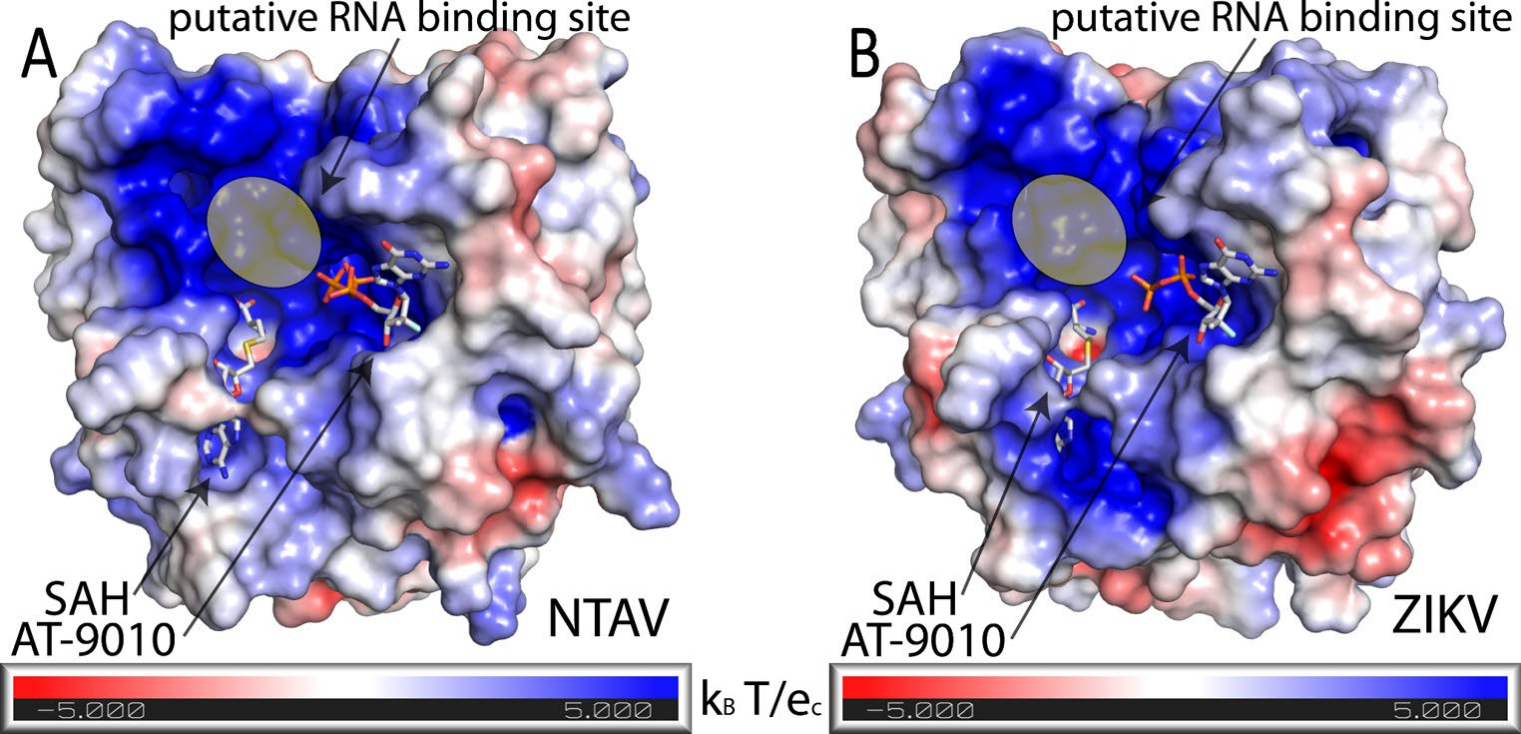
Electrostatic potential visualization of the Ntaya and Zika virus MTase domains in complex with AT-9010 and SAH. (**A** and **B**) The surface of the Ntaya virus (panel A) and Zika virus (panel B) MTase domains is colored according to the electrostatic potential from red (negative charge) to blue (positive charge). AT-9010 and SAH are shown in stick representation and labeled. The putative RNA binding site is highlighted

Both crystal structures had high resolution (2 Å and 1.8 Å), which allowed us to model the ligand precisely and to describe the binding of AT-9010 to both MTase domains in atomic detail (Fig. 2). In the Ntaya virus MTase, the amino group of the guanine ring forms hydrogen bonds with the main chains of Leu16 and Leu19 (Fig. 2A), while in the Zika virus MTase, there is an additional interaction with the main chain of residue Asn17 (Fig. 2B). The 3´-hydroxyl group of the modified ribose ring interacts with main chains of Ser151 and Pro152 and the side chain of Lys13 in the Ntaya virus MTase. However, Lys13 adopts a slightly different conformation in the Zika virus MTase, positioning its amino group 4.5 Å away from the 3´-hydroxyl group of the sugar, thereby not allowing the formation of a hydrogen bond. In addition, the Zika virus MTase has a serine residue at position 152 (Fig. 2D), which does not form a hydrogen bond with the sugar ring, whereas the Pro152 in the Ntaya virus protein does. The α-phosphate in the structure of the Zika virus MTase forms a hydrogen bond with residues Lys28 and Ser227, while, in the case of the Ntaya virus MTase, there is arginine residue at position 28, which does not interact with the phosphate group (Fig. 2). As stated above, in the Ntaya virus MTase structure bound with AT-9010, all three phosphate groups are present, whereas in the Zika virus MTase structure, the γ-phosphate group was not modeled because we did not observe any electron density for it. We suspected that electrostatic interactions might have been responsible for this. However, our analysis revealed that, in both cases, the phosphates are located in a highly positively charged canyon (Fig. 3).

**Fig. 4:**
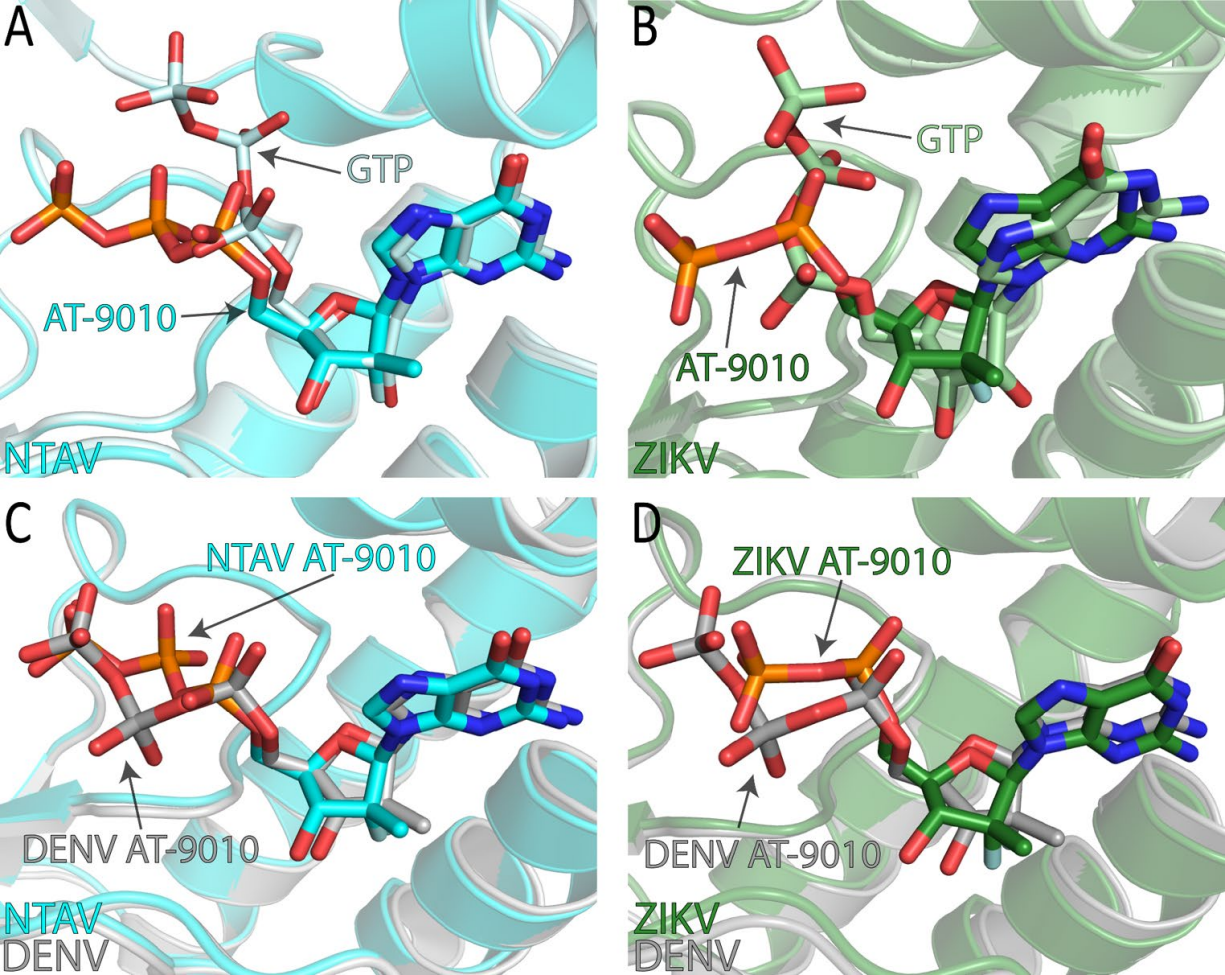
Structural comparison of selected flaviviral MTase domains bound to AT-9010 or GTP. (**A**) Structural alignment of the Ntaya virus MTase domain bound to AT-9010 (cyan) and GTP (pale cyan, PDB ID: 8CQH). The phosphorus atoms of AT-9010 are colored orange, and those of GTP are colored pale cyan. (**B**) Structural alignment of the Zika virus MTase domain bound to AT-9010 (green) and GTP (pale green, PDB ID: 5GOZ). The phosphorus atoms of AT-9010 are colored orange, and those of GTP are colored pale green. (**C**) Structural comparison of the Ntaya (cyan) and dengue virus (gray, PDB ID: 8BCR) MTase domains bound to AT-9010. The phosphorus atoms of AT-9010 interacting with the Ntaya virus MTase are colored orange, and those interacting with the dengue virus MTase are colored grey. (**D**) Structural comparison of the Zika (green) and dengue (gray, PDB ID: 8BCR) virus MTase domains bound to AT-9010. The phosphorus atoms of AT-9010 in Zika MTase structure are colored orange, and those in the dengue virus MTase are colored grey

Next, we compared the binding of AT-9010 in the Ntaya and Zika virus MTases to the binding of GTP (Fig. 4A and B). We observed that the positions of the base, sugar, and α-phosphate are more or less conserved, while the β- and γ-phosphates are flexible. Comparison of AT-9010 binding mode in the Ntaya, Zika, and dengue virus MTases also revealed a conserved position of the sugar and base (Fig. 4C and D).

## Discussion

We observed that the binding mode of AT-9010 is conserved in flaviviral MTases, especially for the base and sugar. The AT-9010 molecule forms hydrogen bonds with key residues at the GTP binding sites of both the Ntaya and Zika virus MTases. However, we noted some differences when comparing the Zika and Ntaya virus MTase structures, where the sugar part of AT-9010 formed significantly more hydrogen bonds with the Ntaya virus MTase than with the Zika virus MTase. In each case, structural comparisons revealed flexibility in the triphosphate portion of the AT-9010 molecule. One contributing factor is residue 28, which may be either lysine or arginine (Fig. 2D). The Zika virus MTase has a lysine at this position that forms a hydrogen bond with the α-phosphate, whereas Arg28 of the Ntaya virus MTase does not. However, overall, the AT-9010 binding mode is sufficiently conserved to provide a structural basis for the function of AT-9010 against multiple orthoflavivirus MTases.

Despite the minor differences described above, such as the γ-phosphate of AT-9010 being highly flexible and not visible in our structure of the Zika virus MTase– contrasting with the visibility of the α-phosphate in a previous structural analysis of the Zika virus MTase in complex with GTP [47]– the overall binding mode of AT-9010 is essentially the same in both enzymes and resembles what has been shown previously for the dengue virus MTase [34]. This conservation provides a structural basis for the broad-spectrum activity of AT-9010 against multiple orthoflavivirus MTases, highlighting its potential as a therapeutic agent targeting various members of this virus genus.

## Electronic Supplementary Material

Below is the link to the electronic supplementary material

Supplementary Material 1

## Data Availability

The structures and related structure factors were deposited in the PDB database under the accession codes 9GJZ (Ntaya virus MTase in complex with AT-9010) and 8PEM (Zika virus MTase in complex with AT-9010).
