# Supplementary material for: Structural basis for broad-spectrum binding of AT-9010 to flaviviral methyltransferases
Source: Arch Virol. 2025 Feb 20;170(3):61. doi: 10.1007/s00705-025-06227-3 (PMC11842469; doi:10.1007/s00705-025-06227-3)
Supplement: Supplementary file 1 — Supplementary Material 1 [file 705_2025_6227_MOESM1_ESM.pdf]

Structural basis for broad spectrum binding of AT-9010 to flaviviral methyltransferases

Katerina Krejcova[1], Evzen Boura[1,*]

[1]Institute of Organic Chemistry and Biochemistry, Academy of Sciences of the Czech Republic, v.v.i, Flemingovo nám. 2, 166 10 Prague 6, Czech Republic.

[*]correspondence to boura@uochb.cas.cz

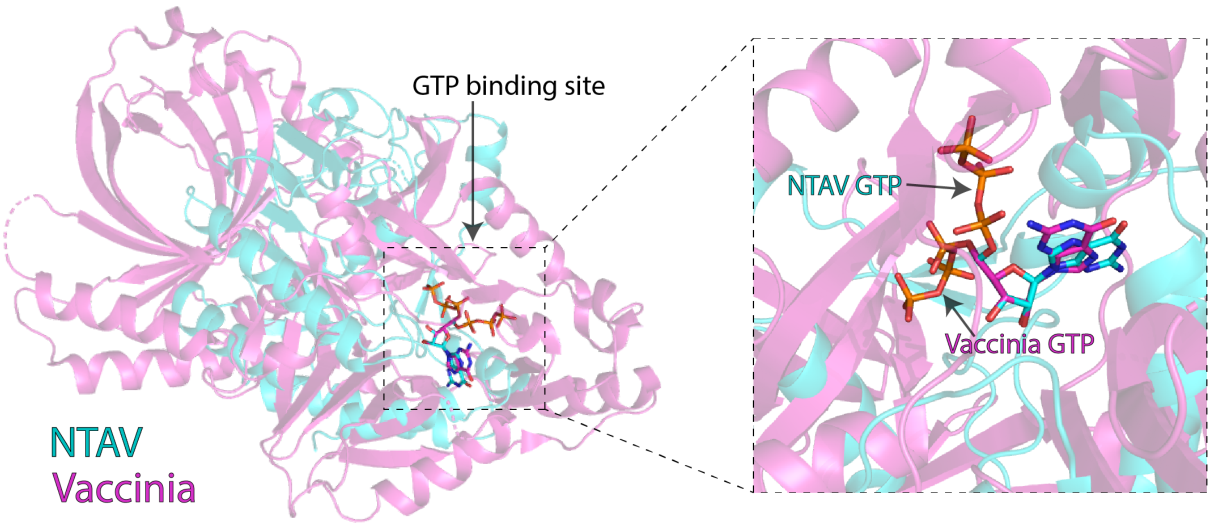

SI Figure 1: Structural comparison of GTP binding sites of Ntaya virus and Vaccinia virus capping enzymes. The structure of the Ntaya MTase domain bound to GTP is shown in cyan (PDB ID:8CQH), while the structure of the Vaccinia virus D1 domain bound to GTP is shown in magenta (PDB ID: 4CKB). Both structures are superposed based on the bound GTP molecule. The right panel provides a zoomed view of the GTP binding sites in both proteins.
